# Political engagement: a key pillar in revitalisation of polio and routine immunisation programmes in the Democratic Republic of the Congo

**DOI:** 10.1136/bmjgh-2024-015675

**Published:** 2024-10-22

**Authors:** Roger Kamba, Amine El Mourid, Raoul Mpoyi Ngambwa, Donat Chungu Salumu, Jean-Bernard Le Gargasson, Daniel Nacoulma, Marcellin Nimpa Mengouo, Nolan Meyer, Christophe Luhata, Nicole A Hoff, Hadia Samaha, Collard Madika, Christelle Mputu, Sylvia Tangney, Cyril Nogier, Chris Diomi, Sydney Merritt, Emma Din, Polydor Kabila, Annabelle Burgett, Didier Nyombo, Emmanuelle Assy, Dalau Mukadi Nkamba, Lora Bertin, Trad Hatton, Didine Kaba, Anne W Rimoin, Elisabeth Mukamba Musenga, Aimé Cikomola, Guillaume Ngoie Mwamba, Sylvain Yuman Ramazani, Kamel Senouci, Magdalena Robert

**Affiliations:** 1 Ministry of Health, Democratic Republic of the Congo, Kinshasa, Congo (the Democratic Republic of the); 2 Bill & Melinda Gates Foundation, Seattle, Washington, USA; 3 McKing Consulting, Kinshasa, Congo (the Democratic Republic of the); 4 Expanded Programme for Immunization, Ministry of Health, Democratic Republic of the Congo, Kinshasa, Congo (the Democratic Republic of the); 5 GAVI the Vaccine Alliance, Geneva, Switzerland; 6 UNICEF, New York, New York, USA; 7 World Health Organization, Geneva, Switzerland; 8 PATH, Democratic Republic of the Congo, Kinshasa, Congo (the Democratic Republic of the); 9 Epidemiology, University of California Los Angeles Jonathan and Karin Fielding School of Public Health, Los Angeles, California, USA; 10 World Bank, Washington, District of Columbia, USA; 11 Development Catalyst, Kinshasa, Democratic Republic of Congo; 12 Ministry of Finance, Democratic Republic of the Congo, Kinshasa, Congo (the Democratic Republic of the); 13 USAID, Washington, District of Columbia, USA; 14 Conseil National de la Courverture Sante Universelle, Ministry of Health, Democratic Republic of the Congo, Kinshasa, Congo (the Democratic Republic of the); 15 Kinshasa School of Public Health, Universite de Kinshasa, Kinshasa, Congo (the Democratic Republic of the); 16 VillageReach, Seattle, Washington, USA; 17 University of Geneva, Geneve, Switzerland

**Keywords:** Health policy, Vaccines, Immunisation, Child health, Global Health

## Abstract

Immunisation is a high priority for improving health outcomes. Yet, in many low-income and middle-income countries, achieving coverage targets independently is hindered by lack of domestic resources and reliance on partners’ support. Both the 2001 Abuja Declaration and 2016 Addis Declaration were key political commitments to improving immunisation coverage; however, many signatories have yet to meet international targets. Despite signing the Global Vaccine Action Plan and Addis Declaration, the Democratic Republic of the Congo (DRC) was unable to fully disburse its portion of allocated funds to cover vaccines without support from Gavi, the Vaccine Alliance and the World Bank between 2017 and 2019. Additionally, during the same time, vaccine coverage outcomes indicated negative trends, with over 750 000 children considered ‘zero-dose’ in 2018. In 2019, a primary focus of the then newly elected President’s agenda was universal healthcare. In collaboration with development partners and stakeholders, the first Presidential Forum was held as a public commitment to increasing childhood immunisation and ensuring the country remains polio-free. This article seeks to highlight the key outcomes of the Forum such as the signing of the Kinshasa Declaration, which formally set targets and specified national, provincial and community-level commitments to vaccination and polio eradication. As of 2023, three Forums have been conducted to reiterate political commitment to routine immunisation in the DRC. This type of high-level commitment could serve as a template for other countries struggling to have high engagement as targets for polio eradication and strengthened routine immunisation are set for 2025–2030.

**Summary box**

- The Democratic Republic of the Congo has seen large increases in governmental and donor funding for routine immunization and polio eradication activities, however, there have been slow changes to vaccination coverage rates.
- This article provides a history of 5 years of high level political engagement in the form of three 'Presidential Forums' in the DRC, including activities leading up to the Forums and outcomes after the Forums.
- This article aims to highlight the high level engagement of political leaders in providing support to routine immunization and polio eradication efforts through bi-annual DRC 'Presidential Forums' which can serve as a framework for health-system improvements in similar settings.

## Introduction

 Since the establishment of the WHO’s Expanded Programme on Immunisation (EPI) in 1974, immunisation has been a cornerstone of public health interventions worldwide.^1^ Historically, many of these programmes in low-income and middle-income countries (LMICs) were funded primarily by international partners.^2^ In these countries, external technical and financial assistance has been critical to maintaining viable immunisation and polio programmes.^2^ Despite this support, many countries have struggled to meet internationally set vaccine coverage targets (such as those set by the Sustainable Development Goals and the Global Vaccine Action Plan) or achieve successful new vaccine introductions.^3^

In general, technical and financial support has been provided for vaccine procurement, new vaccine introduction, operational costs of administering vaccines, supply chain procuration and health and immunisation systems strengthening. To finance vaccine procurement, low-income countries (LICs) have typically relied on Gavi, the Vaccine Alliance, WHO and UNICEF. As a part of Gavi’s financing model, countries receiving vaccines are expected to contribute through cofinancing based on their gross national income per capita.^4^ However, Gavi support is limited to new and underused vaccines only; LIC governments are expected to purchase routine vaccines such as BCG, oral polio vaccine, tetanus-diphtheria and measles-containing vaccine themselves.^5^

Despite these mechanisms to improve access to vaccines in LICs, some countries have struggled to disburse their funding counterparts. In 2001, under the Abuja Declaration, member states of the African Union pledged at least 15% of their national budgets to improving national healthcare systems and delivery.^6^ In 2016, in order to increase domestic resources and encourage sustainable domestic financing for immunisations, 45 African countries agreed to allocate and increase resources for immunisation programmes under the Addis Declaration.^7^ With this commitment, these countries pledged to fund traditional vaccines through domestic resources and increase contributions to their operational costs. While both the Abuja and Addis Declarations were critical steps towards independent immunisation financing, these commitments did not guarantee that individual countries would reach the immunisation targets set.

The Democratic Republic of the Congo (DRC), like many LMICs, has struggled to fund vaccines and operational activities and to meet key vaccine coverage indicators.^3 8^ In 2016, when the DRC was unable to secure government funds, the World Bank (WB) provided funding for traditional vaccines to UNICEF and for Gavi’s cofinancing. The following year, despite having signed the Addis Declaration, the country did not meet its cofinancing obligation on time. In both 2018 and early 2019, the WB provided funding yet again to DRC to cover routine vaccine purchases. Furthermore, prior to 2019, all operational costs related to immunisation activities including vaccine distribution, cold chain, outreach activities and demand-related work in DRC (apart from salaries) were entirely dependent on funding from Gavi, UNICEF, USAID, WB and the UK Department for International Development/Foreign, Commonwealth and Development Office (FCDO).

Regarding vaccine coverage indicators, both the 2013–2014 Demographics and Health Survey and the 2018 Multiple Indicator Cluster Survey (MICS) indicated poor immunisation coverage nationally–with reported national diphtheria-tetanus-pertussis-3 (DTP3) coverage at 60.5% and 47.6%, respectively.^9 10^ Results from the 2018 MICS indicated that all but 1 of the 26 provinces had less than 50% full immunisation coverage among children 12–23 months old.^10^ Additionally, the 2018 MICS estimated that over 1.1 million children living in DRC were considered zero-dose—defined by Gavi as never having received a dose of DTP1.^10 11^ In response to these low immunisation rates, the Ministry of Health, in collaboration with a number of partners, implemented an emergency plan for the revitalisation of the routine immunisation system called the Mashako Plan.^12^ This plan was focused on programmatic activities including improvements to the cold chain, supervision, service delivery, vaccine supply, real-time monitoring of output indicators, as well as regular vaccine coverage surveys (VCS) for the routine immunisation programme in nine initial provinces.^12^ While the Mashako Plan focused on programmatic improvements with key roles from political leadership, formalised political commitment at the highest level was the missing component to ensure the success of the transformation plan. This article expands on the targeted efforts to codify political commitments to the Mashako Plan through the establishment of a National Presidential Forum in DRC.

### Implementing a National Presidential Forum

In 2019, the newly elected President, H.E. Félix Antoine Tshisekedi Tshilombo, announced his vision for a new social agenda that included free education and universal healthcare. To set the political groundwork in support of the improvement of immunisation and polio eradication, the presidency, with immunisation partners, organised the ‘National Forum in Support of Vaccination and the Eradication of Polio’ (The Presidential Forum).^12^ The primary goal of The Presidential Forum was to increase political involvement, motivation, domestic and international funding, as well as accountability for polio eradication and increasing childhood immunisation through government engagement and ownership, both at the national and subnational levels.

The first Presidential Forum was held in Kinshasa on 22 July 2019 and 23 July 2019, with support from immunisation partners (WHO, UNICEF, Gavi, CDC, USAID, FCDO, Rotary, WB, Bill & Melinda Gates Foundation (BMGF)) and technical assistance partners (PATH, Acasus, John Snow (JSI), VillageReach, SANRU, International Medical Corps, Kinshasa School of Public Health and University of California, Los Angeles). Additionally, to publicly communicate the Presidential commitment to the international community, a multinational, representative cohort which included the WHO AFRO Regional Director, Gavi CEO, WB Head of Health, Rotary President, the BMGF President of Global Development and representatives of the Center for Disease Control, Atlanta, as well as ambassadors from donor countries were invited to participate. Delegations from all 26 provinces included the Governor, the President of Provincial Assembly, as well as Provincial Ministers of Health and the Chiefs of Health.

The 2-day Forum opened with speeches from the President of the National Assembly, the Special Advisor to the President charged with universal health coverage, international partners, the National Minister of Budget, and finally the President of the Republic. In addition, the President participated in high-level round table discussions with the leads of international agencies to discuss common goals for improving routine immunisation, polio and the role of the government. At the conclusion of these meetings, the President, ministers, governors of each province and presidents of provincial assemblies signed the Declaration of Kinshasa on Routine Immunisation and Polio Eradication. The national immunisation programme also held round tables with provincial delegates to discuss specific commitments by each province. During these high-level round tables, the President requested support from international partners in monitoring the commitments of the Declaration. Lastly, this Forum included the first biannual review of the Mashako Plan: the top three performing provinces and districts were awarded ‘Champions of Vaccination’ certificates by the Secretary General of Health.

The Declaration of Kinshasa formally committed national-level, provincial-level and community-level leaders to improving childhood immunisation rates. A publicly available website in both French and English was created to track adherence to the main commitments of the Declaration.^13^ Both national and provincial governments made specific commitments (table 1), of which the most critical are as follows:

- National: Timely and complete disbursement for the purchase of traditional vaccines and cofunding for Gavi-supported vaccines.
- National: Quarterly monitoring of the Declaration of Kinshasa by the Prime Minister during the Government council, monthly monitoring of the Kinshasa Declaration by the Minister of Health.
- Provincial: Provincial law to fund health and immunisation activities.
- Provincial: Target of US$1/child (for children aged 0–11 months) from provincial funds to support operational costs of immunisation.
- Provincial: Dedicated follow-up of vaccination activities through quarterly review of immunisation activities and commitments to hold quarterly meetings with the governor (CPP), monthly meetings and field monitoring of vaccination sessions with the Provincial Minister of Health (CCIA) and creating a network with government parliament for vaccination support (REPCAV).

**Table 1:** Vaccine milestones targeted in the Kinshasa Declaration: 2018–2022

	2018	2019	1st Presidential Forum	2020	2021	2022
Vaccine disbursements (in million USD)	4.4	9		16.4	17.8	18.2
Provincial ministry meetings	--	--		8/26	10/26	5/26
Number of provinces conducting the minimum number of governor’s meetings	--	--		5/26	10/26	9/26
# Provinces with a law to fund immunisation activities	--	--		16/26	18/26	18/26
# of Provinces contributing any financing towards immunisation	--	--		7/26	9/26	7/26
# of Provinces contributing US$1 or more per child for vaccine activities	--	--		--	3/26	2/26
Provincial contribution to Immunisation activities (>US$100 000)	0	0		0	4	2
DTP3 coverage (# of provinces over 80%)	1/26	--		3/18	2/26	4/26
Provinces under the Mashako Plan	9/26	9/26		18/26	18/26	26/26
Full immunisation coverage (# provinces over 60%)	1/26	--		5/18	3/26	5/26
# of provinces with estimates of ‘zero-dose’* children under 10%	1/26	--		3/18	7/26	6/26
# of provinces with cVDPV2 or cVDPV1 cases	6/26	12/26		10/26	4/26	14/26

* Zero-dose is defined by Gavi as 100-Pentavalent-1 coverage.

cVDPV2, cases of confirmed vaccine-derived poliovirus type 2; DTP3, diphtheria-tetanus-pertussis-3.

**Figure 1:**
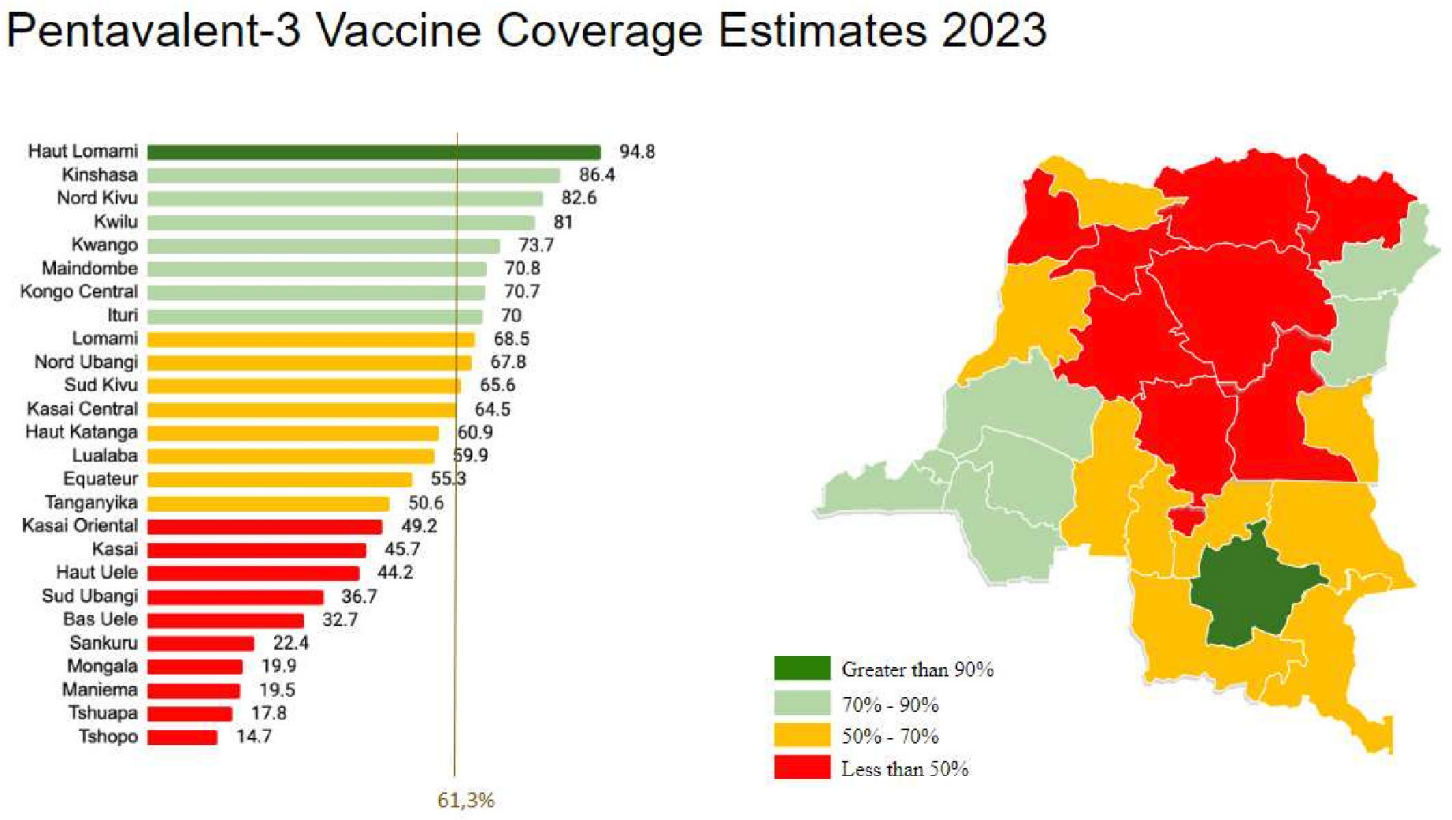
Pentavalent-3 vaccince coverge estimates for the 26 provinces for 2023 in descending order - highest to lowest percentage.

Beyond the public website, the government and partners agreed to a scorecard mechanism to monitor adherence to the main commitments of the declaration. These scorecards were generated monthly for each province by health zone and included a summary of all EPI monthly supervision data to determine how well individual provincial services were performing. The declaration commitments, as well as comparisons of vaccine coverage estimates between provinces, were reviewed at each subsequent Forum (figure 1).

Initially, the Forums were to be conducted on an annual basis to track progress and renew political commitments at the highest level. In practice, due to the COVID-19 pandemic and the complexity of organisation, the Forums were held every 2 years. Following the first Forum’s success, the second Presidential Forum was held in Kinshasa on 21 October 2021. The government organised a review of provincial routine immunisation and polio progress under the Mashako Plan including recognition of improvements in immunisation coverage and a review of the main commitments of the Kinshasa Declaration. In addition, the President made a speech recommitting the government to the same immunisation targets, confirming continued government support of his social welfare agenda and the Kinshasa Declaration, and ensuring all children in the DRC have access to routine vaccinations. The second Forum presented results which indicated immediate improvements in vaccination rates. For example, the national pentavalent-3 coverage rates increased by 20 percentage points between the 2018 MICS (47.6%) and the 2020 VCS) (67.6%). This Forum also marked the first discussions on the Mashako Plan 2.0—an expansion of the existing plan with additional priorities and to all DRC provinces.

The third Presidential Forum was held in Kinshasa on 27 June 2023 and 28 June 2023. Beyond the establishment of the Presidential Forum as a regular biannual event, notable milestones of the third Forum included the integration of all 26 provinces under the Mashako Plan, and the disbursement of US$2.3 million by provinces for vaccination since the signing of the Kinshasa Declaration. Additionally, the Forum included the presentation of the latest data from the VCS conducted in March–April 2023, showing some coverage improvements from COVID-19-pandemic backsliding. The participants attended special sessions on ending polio outbreaks, reaching zero-dose children, improving Mashako Plan indicators and increasing domestic financing for immunisation. The Minister of Health gave prizes to the best performing and most improved provinces. The Secretary General of Health also held a panel with governors of the best and lowest-performing provinces, based on the results of the coverage surveys. Several governors committed to reinvigorating their immunisation programmes and monitoring progress more regularly.

### Patient and public involvement

Neither patients nor the public were involved in the activities presented in this article.

### Impacts of the Presidential Forums

**Figure 2:**
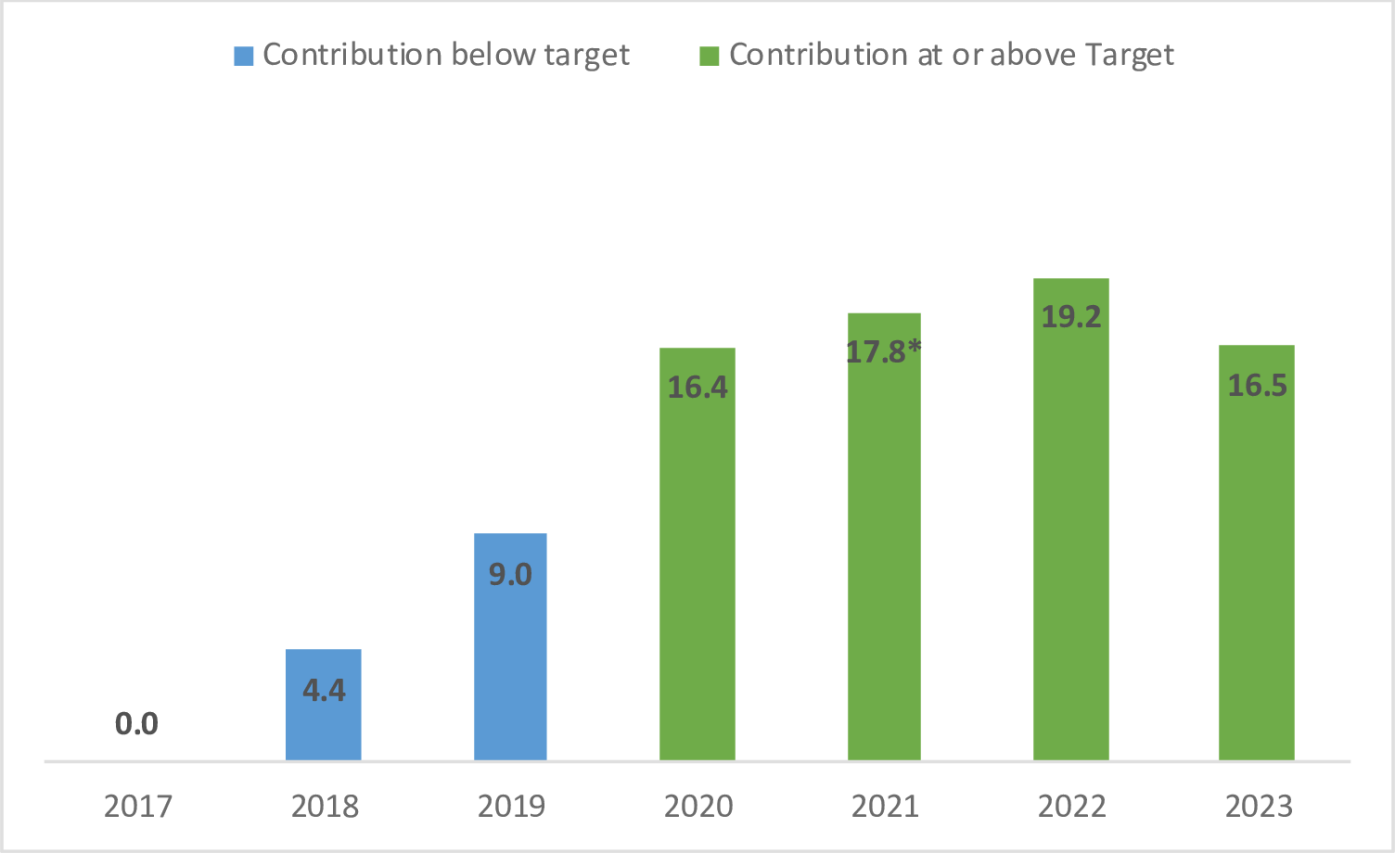
Yearly contributions of the DRC National Government to funding vaccination contributions (in millions, USD) from 2017-2023.

These Forums directly promoted high-level commitment to vaccination and played a role in changing the processes and increasing domestic sources from which routine immunisation programmes are funded in the DRC. Prior to the implementation of the Mashako Plan, routine immunisation in the DRC had been almost entirely funded and supported by international development partners. With the implementation of the Mashako Plan and public presidential support, provincial governments themselves were encouraged to contribute directly to their immunisation programmes. By 2021, 8 of the 26 DRC provinces were contributing to financing their own vaccination programmes (table 1). At the highest level of DRC governance, these Presidential Forums were instrumental in encouraging provincial buy-in and participation in the routine immunisation programmes of a decentralised state. Furthermore, following the first Forum, and for the past 4 years, the national government fully funded its vaccine commitments in 2020, 2021, 2022 and 2023 (US$17–US$21 million per annum) (figure 2). The country also joined the UNICEF Vaccine Independence Initiative which provides more financial flexibility to ensure an uninterrupted supply of vaccines. In addition, the new International Monetary Fund programme 2021–2023 included disbursement targets for vaccines as part of the social spending targets. The Presidential Forums and the commitments made by the government were key to ensure improved financing for vaccines.

Another direct effect of the Presidential Forums was the increased availability of funding for operational activities by provinces—18 provinces signed edicts for funding immunisation at the provincial level between the first and second Forum. Over the course of the 5 years following the first Presidential Forum, all 26 provinces have increased funding for day-to-day activities of immunisation services and delivery (table 1).

The Presidential Forums were an important element in motivating provincial leaders to adhere to their signed commitments under the Kinshasa Declaration and anecdotally may have created competition between provinces to complete timely payments and strive to increase vaccination coverage. As shown in table 1, under the six primary commitments: vaccine budgeting, provincial funding, and monitoring activities are currently considered ‘on track’. While ministry meetings, full immunisation coverage and governor meeting counts have not yet met their target, these indicators have improved over time following the initial signing of the declaration. Specifically, vaccine financing improved over time as the DRC government and partners collaborated with the International Monetary Fund to include purchasing contributions by the government as a condition of larger disbursements. In provinces, increased contributions can be attributed to partnerships with donors where basket funds were established to codisburse immunisation activities at the subnational level.^14^

As the Presidential addresses were most immediately directed at provincial leaders and international partners, they are not directly responsible for individual-level changes in vaccine demand or coverage. Following the first Presidential Forum, rapid increases in coverage were observed in almost all provinces of the DRC. These increases were observed between the pre-Forum 2018 MICS and later vaccine coverage surveys in 2020 and 2021 for pentavalent-1, pentavalent-3, measles and full-immunisation coverage (table 2). While these increases were initially significant, the continued improvement of vaccine coverage was not sustained due to national vaccine shortages, stockouts, the COVID-19 pandemic and healthcare worker strikes. Vaccine coverage was also bolstered by the introduction of the Mashako Plan in 2018 as a programmatic intervention to improve coverage rates. The most recent vaccine coverage survey estimates indicate that, while coverage did not drop below pre-Forum estimates, the dramatic improvements following the first Forum were not sustained, instead, vaccine coverage only mildly improved from the pandemic-era decrease. Preliminary estimates from the 2023 VCS indicate a minimal increase nationally overall, with some individual provinces demonstrating dramatic improvements in coverage. Immunisation coverage in Haut Lomami, for example, increased from 35.7% in the MICS 2018 estimate to 88.9% in the most recent VCS. Reported pentavalent-3 coverage in this province also increased from 50.4% to 94.8% over the same period (figure 1).

**Table 2:** National vaccine coverage 2018–2022 from various representative surveys

Vaccine	MICS 2018 (n=4287)	1st Presidential Forum	VCS 2020 (n=48 093)*	VCS 2021–2022 (n=88 592)	VCS 2023 (n=47 880)
Full immunisation†	35.0%		52.5%	41.5%	45.3%
Penta 1‡	65.8%		83.2%	80.9%	81.2%
Penta 3‡	47.6%		67.6%	60.3%	61.3%
Measles (MCV)	57.2%		68.5%	55.9%	56.1%

* VCS 2020 only included data from 18 provinces, not all 26 provinces of the DRC.

† Full immunisation indicates the percentage of children who have received all routine immunisation doses (as prescribed by the DRC Ministry of Health).

‡ Penta1 and Penta3 indicate the first and third doses of the DTP-containing pentavalent vaccine.

DRC, Democratic Republic of the Congo; DTP, diphtheria-tetanus-pertussis; MCV, measles-containing vaccine; VCS, vaccine coverage survey.

The Presidential Forum’s goal was also to increase focus on polio eradication efforts. Following the subnational commitments at the first Forum, cases of confirmed vaccine-derived poliovirus type 2 (VDPV2) decreased in some provinces, namely Haut Lomami and Tanganyika. However, as of 2022, there has been a surge in VDPV2 cases as well as identification of VDPV1 cases in several provinces (table 1). Following the spike in cases observed in 2022, the national government announced an increased focus on outbreak response activities. At the provincial level, the President demanded during the Forums that governors make polio-outbreak response a priority.

### Forum successes and limitations

The Forums in DRC succeeded in increasing focus, ownership and participation in immunisation activities at the highest levels of national and provincial government (table 3). By focusing on high-level financial and vaccine coverage targets, the declaration and subsequent monitoring mechanisms focused the attention of leaders on critical activities. With the increased oversight of the President and national attention, another positive outcome of this political advocacy was the improvement in disbursement for vaccines, with the country fulfilling its financial obligations 4 years in a row—the first time in its history.

**Table 3:** Major outcomes of each Presidential Forum (2021–2023)

Presidential Forum	Major accomplishments
Forum 1–2019	Commitment of the central government to fund vaccine purchases on time Commitment that each province will fund US$1 per child for operational costs Commitment to establish regular reviews with governors to evaluate routine immunisation and polio eradication progress
Forum 2–2021	Expansion of the Mashako Plan to include all 26 provinces and rapid increase in coverage among the original nine provinces
Forum 3–2023	Launch of the equity accelerator fund to target zero-dose children and increased focus on provinces with poor routine immunisation performance DRC President sets target for the country to reach 75% coverage by 2027

DRC, Democratic Republic of the Congo.

However, the most recent survey results indicate a stagnation—following the initial increase in 2020—of coverage improvements which can be attributed to factors such as healthcare worker strikes, and supply-chain difficulties due to the COVID-19 pandemic. While the most recent results included are from the 2023 VCS, these coverage estimates include children up to 24 months old, who would have been affected by events up to 2 years preceding data collection. Additional efforts are required to improve vaccination performance.

Overall, the Presidential Forums are one part of a multifaceted effort to revitalise routine immunisation in DRC. These Forums did not serve as formal evaluations, but instead as motivators at the highest levels of government for subnational leadership of routine immunisation with the support of international development partners. Direct outcomes of the Presidential Forums in DRC have been increasing financial disbursements from each province under the Mashako Plan, and national commitment to the full funding of the vaccine stock and the signed Kinshasa Declaration, which ensured that tangible results and commitments were enforced following the Forums. While not every LIC may need the same type of support from their national government, this type of public activity in DRC has been well received and could serve as a template for other countries that have not yet met their pledges from the 2016 Addis Declaration.

## Conclusion

Presidential Forums serve as an example of how high-level political support at both the national and subnational levels associated with a measurable accountability framework can improve public health programme outcomes. However, they should not be seen as the only way LICs can achieve high vaccination coverage, instead, they should be seen as an opportunity for countries to engage high-level political leaders with the task in mind to increase political visibility and political support. With this in mind, biannual Forums were a key tool to support routine immunisation and polio eradication in DRC. This approach to complement programmatic support could be replicated in other settings as an additional mechanism for public health advocacy.

## Data Availability

Data are available in a public, open access repository.
